# Antibacterial Effect of Silver Nanoparticles against Oral Biofilms in Subjects with Motor and Intellectual Disabilities

**DOI:** 10.3390/jfb15070191

**Published:** 2024-07-10

**Authors:** Carolina Holguín-Meráz, Rita Elizabeth Martínez-Martínez, Erasto Armando Zaragoza-Contreras, Rubén Abraham Domínguez-Pérez, Simón Yobanny Reyes-López, Alejandro Donohue-Cornejo, Juan Carlos Cuevas-González, Erika de Lourdes Silva-Benítez, Nelly Molina-Frechero, León Francisco Espinosa-Cristóbal

**Affiliations:** 1 Master Program in Dental Sciences, Stomatology Department, Institute of Biomedical Sciences, Autonomous University of Juarez City (UACJ), Envolvente del PRONAF and Estocolmo s/n, Ciudad Juárez 32310, Chihuahua, Mexico; al220733@alumnos.uacj.mx (C.H.-M.); adonohue@uacj.mx (A.D.-C.); juan.cuevas@uacj.mx (J.C.C.-G.); 2 Master Program in Advanced Dentistry, Faculty of Dentistry, Autonomous University of San Luis Potosi, Manuel Nava Avenue, University Campus, San Luis Potosí 78290, San Luis Potosí, Mexico; ritae_martinez@hotmail.com; 3 Department of Engineering and Materials Chemistry, Centro de Investigación en Materiales Avanzados, S. C., Miguel de Cervantes No. 120, Chihuahua 31136, Chihuahua, Mexico; armando.zaragoza@cimav.edu.mx; 4 Laboratory of Multidisciplinary Dental Research, Faculty of Medicine, Autonomous University of Queretaro, Clavel Street, Prados de La Capilla, Santiago de Querétaro 76176, Querétaro, Mexico; dominguez.ra@uaq.mx; 5 Institute of Biomedical Sciences, Autonomous University of Juarez City (UACJ), Envolvente del PRONAF and Estocolmo s/n, Ciudad Juárez 32310, Chihuahua, Mexico; simon.reyes@uacj.mx; 6 Faculty of Odontology, Autonomous University of Sinaloa, Josefa Ortiz de Domínguez Street, Culiacán 80010, Sinaloa, Mexico; erikasilva@uas.edu.mx; 7 Division of Biological and Health Sciences, Autonomous Metropolitan University Xochimilco (UAM), Mexico City 04960, Mexico; nmolinaf@hotmail.com

**Keywords:** nanoparticles, silver, anti-bacterial agents, persons with disabilities, dental plaque, biofilms

## Abstract

Background: Motor and intellectual disabilities (MIDs) represent a great challenge for maintaining general health due to physical and cognitive limitations, particularly in the maintenance and preservation of oral health. Silver nanoparticles (AgNPs) have emerged as a promising therapeutic tool for bacterial control, including oral biofilms; however, knowledge of the bactericidal effectiveness of oral biofilms from patients with MIDs is insufficient. This study aims to determine the antimicrobial effect of AgNPs on different oral biofilms taken from patients with and without MIDs. Methods: Two sizes of AgNPs were prepared and characterized by dynamic light scattering (DLS) and transmission electron microscopy (TEM). Through consecutive sampling, biofilm samples were collected from 17 subjects with MIDs and 20 subjects without disorders. The antimicrobial effect was determined by obtaining the minimum inhibitory concentration (MIC) of AgNPs, and the identification and distribution of oral bacterial species were determined by polymerase chain reaction (PCR). Finally, correlations between sociodemographic characteristics and the antimicrobial levels of AgNPs were also explored. The values of the MIC results were analyzed with IBM-SPSS software (version25) using non-parametric tests for independent groups and correlations, with statistical significance being considered as *p* < 0.05. Results: Both sizes of AgNPs exhibited tight particle size distributions (smaller: 10.2 ± 0.7 nm; larger: 29.3 ± 2.3 nm) with zeta potential values (−35.0 ± 3.3 and −52.6 ± 8.5 mV, respectively) confirming the stability that resulted in little to no agglomeration of nanoparticles. Although both sizes of AgNPs had good antimicrobial activity in all oral biofilms, the smallest particles had the best antimicrobial effects on the oral biofilm samples from patients with and without MIDs, even better than chlorhexidine (CHX) (*p* < 0.05). Likewise, the patients with disabilities showed higher levels of antimicrobial sensitivity to AgNPs compared with CHX (*p* < 0.05). Although the microorganisms included in the biofilms of females had a statistically higher growth level, the AgNP antimicrobial effect was statistically similar in both genders (*p* > 0.05). The most frequent bacteria for all oral biofilms were *S. mutans* (100%), *P. intermedia* (91.6%), *T. forsythia* (75.0%), *T. denticola* (75.0%), *P. gingivalis* (66.6%), *F. nucleatum* (66.6%), *S. sobrinus* (50.0%), and *A. actinomycetemcomitans* (8.3%). Conclusions: AgNPs exhibited considerable antimicrobial potential to be used as a complementary and alternative tool in maintaining and preserving oral health in patients with MIDs.

## 1. Introduction

Disability is defined by the World Health Organization (WHO) as a permanent or transitory condition that humans experience at some specific points in their lives, limiting regular activities, such as seeing, hearing, walking, remembering or concentrating, performing self-care, and communicating with others, and affecting 16% of persons worldwide (~1.3 billion people) [1]. This population experiences relevant health inequalities, stigmatization, discrimination, poverty, and exclusion from education and employment, among others, in comparison with people without disabilities [1,2]. Additionally, oral diseases are still considered a severe oral health problem in the world, estimated to affect almost 350 million people (45%) [3]. Dental caries and periodontal disease represent the most prevalent infectious and multifactorial oral diseases, with the former affecting 200 million adults and 514 million children and the latter affecting more than 100 million adults [3]. Particularly, patients with disabilities suffer poor oral health and need additional support to maintain adequate oral care [4], resulting, basically, in relevant oral health inequalities [5]. Authors have reported that patients with disabilities, especially intellectual disorders, present a higher prevalence of oral diseases, especially periodontal disease and dental caries [6]. It is worth noting that in these patients, this condition also affects their general health, facilitating the presence of cardiovascular disease, diabetes, respiratory disease, and strokes, to the detriment of their quality of life [7]. 

It is very well known that oral biofilms represent a crucial etiological factor involved in the beginning and development of dental caries and periodontal disease, resulting in a significant oral health challenge for dentistry [8]. At the same time, the antibiotic resistance of important pathogenic bacteria has resulted in severe health problems worldwide [9,10]. Several oral bacteria related to dental caries and periodontal disease have been demonstrated to be resistant to antimicrobials considered as gold standard agents in dentistry [11,12]. The oral biofilm comprises very complex and well-organized bacteria that adhere to tooth surfaces and dental materials, covered by an extracellular matrix that facilitates linkages between bacteria species and solid surfaces [13]. Extracellular polysaccharides, such as glucosyltransferase (GTF) and fructosyltransferase (FTF), are the agents responsible for promoting bacterial adhesion to teeth, resulting in well-conformed biofilms [14,15]. Reports have suggested that the presence and distribution of particular bacterial strains depend on the type of biofilm. Other studies have determined the specific bacterial distribution and the worst clinical aspects among subjects with disabilities, concluding that the microbiota compositions of specific oral biofilms from patients with MIDs are different from those of subjects without disabilities [16,17]. The above information probably explains why conventional oral treatments, based on non-surgical periodontal treatment and focused on controlling periodontitis (the most severe of the periodontal disease), have not been able to reduce bacterial populations in subjects with Down syndrome (DS) when compared with controls [18]. Furthermore, chlorhexidine gluconate (CHX) has been considered by many studies as the main antimicrobial agent in dentistry and is positioned as the gold standard solution; however, the pigmentation of dental tissues and tongue, mucosal irritation, xerostomia, numbness, and other disturbances have been associated with the presence and chronic use of CHX [19]. Consequently, it is necessary to explore new and novel antimicrobial approaches for controlling and preventing dental caries and periodontal disease in patients with disabilities. 

Some antimicrobial materials have been designed for biomedical applications to control bacteria that often become resistant to conventional antimicrobials. Different types of coatings using a combination of calcium acetate, hydroxyapatite, and zinc oxide or even copper-based metallic glass composites have offered good or even excellent antibacterial activities against different types of microorganisms, including methicillin-resistant ones [20,21]. Silver nanoparticles (AgNPs) have been extensively recommended because of their excellent antimicrobial effects against various microbial species [22,23], including oral bacteria [24,25]. Several works have determined that these metallic nanostructured materials control and prevent biofilm formation, resulting in high antimicrobial and antibiofilm potential for dental applications [25,26,27,28]. Also, studies have indicated that AgNPs have antifungal properties against *Candida* species isolated from patients with stomatitis [27], considering AgNPs’ capacity to reduce and control biofilm formation [27,29], principally for *Candida albicans* and *Candida parapsilosis* [27]. Another investigation on the antimicrobial and antibiofilm activities of AgNPs using plant extract-mediated synthesis on bacteria related to dental caries, such as *Streptococcus sanguinis* (*S. sanguinis*), *S. mutans*, *Lactobacillus acidophilus* (*L. acidophilus*), and *C. albicans*, showed that AgNPs exhibited antimicrobial activity against all bacterial species and also inhibited the biofilm formation of *S. mutans* on the surface of tooth enamel [25]. 

To the best of our knowledge, there is no scientific information that has determined the bacterial inhibition growth by AgNPs from oral biofilms related to patients with mental and physical incapacities. This study aimed to investigate the antimicrobial activity of AgNP with two different particle sizes against oral biofilms taken clinically from patients with and without MID. Additionally, we explored the associations and correlations between the bacterial inhibition effect of AgNPs against the sociodemographic and clinical peculiarities of each patient. This study will improve the understanding of the safe and predictive use of AgNP for the control and prevention of oral diseases derived from bacterial accumulation in patients with MID, as an alternative antimicrobial tool for daily oral health maintenance.

## 2. Materials and Methods

### 2.1. Synthesis and Characterization of AgNPs

Two families of AgNPs were prepared following the method previously reported [26]. For the first type, 0.01 M of silver nitrate (AgNO_3_, CTR Scientific, Monterrey, Mexico) was dissolved in 100 mL of deionized water for 5 min under magnetic stirring in a 250 mL glass container. Afterward, 10 mL of deionized water with 0.1 g of gallic acid (C7H_6_O_5_, Sigma Aldrich, St. Louis, MI, USA), which was used as a reducing agent, was added to the solutions, then the pH was immediately adjusted to 11 with a 1.0 M solution of sodium hydroxide (NaOH, Jalmek Scientific, San Nicolás de los Garza, Mexico). Similar concentrations of AgNO_3_ were used for the second solution, while the amount of gallic acid was increased to 0.5 g. Finally, the pH was adjusted to 10 with ammonium hydroxide (NH_4_OH, molecular weight 35.05, catalog A5325, number CAS: 1336-21-6, Jalmek Scientific, San Nicolás de los Garza, Mexico). Sodium and ammonium solutions were used to stabilize the particle size for each AgNP sample, respectively. Both samples continued to be stirred for 10 min at room temperature. The average particle size, particle size distribution, and electrical properties of AgNPs were determined by dynamic light scattering using a nanoparticle analyzer (DLS, Nanoparticle Analyzer, Nano Partica SZ-100 series, HORIBA Scientific Ltd., Irvine, CA, USA). The particle shape of AgNPs was analyzed using transmission electron microscopy (TEM, Phillips CM-200, Philips Electronics NV, Eindh, Netherlands) in a voltage accelerating of 25 kV. 

### 2.2. Patient Recruitment

The patients were recruited from the Multidisciplinary Clinic of the Stomatology Department of the Institute of Biomedical Sciences (ICB) at the Autonomous University of Juarez City (UACJ), using consecutive non-probabilistic sampling, between January and December 2019. A written and voluntarily signed informed consent was individually obtained from each patient or legal guardian of the patient’s previous sample collection, following the ethical guidelines of the Helsinki Declaration (2008). This project was approved by the Bioethics Committee of the ICB-UACJ (project ID RIPI2022ICB10). This study included 37 subjects between 10 and 27 years old who had brushed their teeth at least three hours before taking samples. The subjects were divided into two groups as follows: (a) patients with motor and intellectual disorders, and (b) patients without motor and intellectual disorders (healthy group). A physician or legal guardian of each patient confirmed the presence and the type of MID. Subjects with unclear or undefined medical diagnosis, systemic compromise, and those who received periodontal therapy or took antibiotics within the last three months were excluded. 

### 2.3. Collection of Oral Biofilms 

Oral biofilms were obtained using mechanical sweeps with sterilized wooden toothpicks in the gingival sulcus (subgingival level) and the gingival margin (supragingival level) of interproximal sites from lower posterior teeth. Subsequently, the toothpicks were immediately cultured in Müller–Hinton broth (MH, BD™ Difco™, Rockville, MD, USA) and incubated for anaerobic bacteria at 37 °C for 24 h. 

### 2.4. Bacterial Growth Rate and Standard Bacterial Suspension

The bacterial growth rate was determined after 24 h of initial incubation and previous antimicrobial tests. First, 100 µL of each bacterial suspension was dispersed in a cell containing 3 mL of phosphate buffer solution (PBS, pH 7.4). Then, the absorbance level was analyzed by spectrometry (Eppendorf BioPhotometer Plus, Hamburg, Germany) using a wavelength of 550 nm in triplicate. Once the initial bacterial growth rate was determined, a standardized bacterial suspension containing 1.5 × 10^8^ colony-forming units per milliliter (CFU/mL) was prepared according to the McFarland scale. Finally, each bacterial suspension was diluted until it reached a concentration of 1.5 × 10^6^ CFU/mL, which was used for all antimicrobial assays. 

### 2.5. Antimicrobial Test of AgNPs on Oral Biofilms 

The antibacterial assay used in this work was based on the previously reported protocol [26]. All oral biofilm samples were cultured in MH broth for 24 h at 37 °C before testing. Minimum inhibitory concentrations (MICs) were determined by incubating each bacterial sample exposed to different antimicrobial treatments using 96-well microdilution plates with 100 µL of MH broth in each well. Initially, 200 μL of each AgNP dispersion was placed on the first column, and serial dilutions were made until the eleventh. Then, 100 µL of each standardized bacterial suspension, containing approximately 1.5 × 10^6^ CFU/mL, was placed in all columns. Finally, the microdilution plates were stored and incubated at 37 °C for 24 h. The first and twelfth columns were used as the negative (no bacterial growth) and positive (bacterial growth) controls, respectively. The MIC values were determined through turbidimetry using visual and stereoscopy examinations in the last well with bacterial growth comparing the positive and negative control columns. The 2% chlorhexidine solution (CHX, Consepsis™, Ultradent, Tokyo, Japan) was used as a gold standard using similar parameters for the antimicrobial assay. All antimicrobial tests were performed in triplicate. Finally, microscopic evaluation using scanning electron microscopy (SEM, JEOL JSM-6510, JEOL Ltd., Tokyo, Japan) was used to explore the bactericidal and antibiofilm activity of the AgNPs in a reference strain of *Streptococcus mutans* (*S*. *mutans*, ATCC 25175). 

### 2.6. Identification of Bacterial Species by Polymerase Chain Reaction (PCR) 

Twelve oral biofilms from young male and female patients nearly 15 years old with (*n* = 8 subjects) and without (*n* = 4 subjects) disabilities were randomly selected for the identification and distribution of several microbial species related to dental caries and periodontal disease using the polymerase chain reaction (PCR) assay. The occurrence of *Streptococcus mutans* (*S. mutans*), *Streptococcus sobrinus* (*S. sobrinus*), *Porphyromona gingivalis* (*P. gingivalis*), *Tannerella forsythia* (*T. forsythia*), *Treponema denticola* (*T. denticola*), *Prevotella intermedia* (*P. intermedia*), *Fusobacterium nucleatum* (*F. nucleatum*), and *Aggregatibacter actinomycetemcomitans* (*A. actinomycetemcomitans*) were determined using previously reported methods [30,31] and specific primers [32,33,34,35,36,37,38]. Positive and negative controls were included for each PCR assay. All products were processed in 2% agarose gels by electrophoresis, stained, and analyzed with UV light (E-Gel Imager System with UV Base, Thermo Fisher Scientific, Life Technologies, Waltham, MA, USA).

### 2.7. Statistical Analysis

The patients’ sociodemographic variables according to gender were expressed in frequency and percentage. The values of age, size distribution, zeta potential, bacterial growth rate, and MIC level were defined using descriptive statistics expressed in mean and standard deviation. The homogeneity in the study groups was analyzed using Pearson’s chi-square test and the student’s *t*-test. The Shapiro–Wilk analysis was used to define the distribution of the variables for independent groups. The Mann–Whitney U test was used to determine the differences between independent groups for non-parametric variables. Additionally, Spearman’s rho test was used to analyze significant correlations between age and the bacterial growth rate and between antimicrobial activity levels of AgNPs. IBM-SPSS software (SPSS, version 25, Chicago, CA, USA) was used for all statistical tests determining a statistical significance when *p* < 0.05.

## 3. Results

### 3.1. Characterization of AgNPs

**Figure 1:**
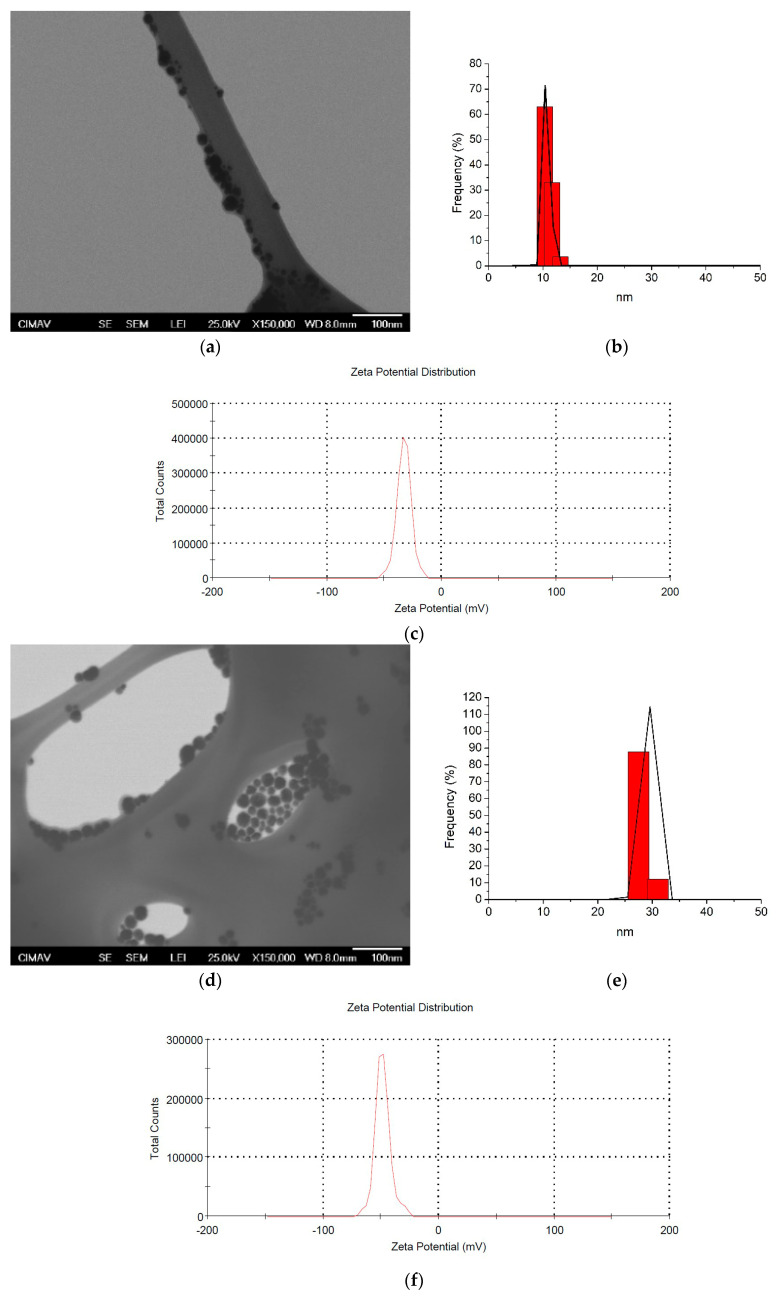
Characterization of AgNPs by DLS and TEM. (**a**,**b**) 10.2 nm, ×150,000; (**d**,**e**) 29.3 nm, ×150,000; (**c**,**f**) zeta potential images (10.2 and 29.3 nm, respectively).

Table 1 shows the physical characterization of the AgNPs. Two different sizes of AgNPs were obtained, a small (10.2 ± 0.7 nm) and a larger one (29.3 ± 0.7 nm), with a spherical shape. DLS showed single- and narrow-based peaks, suggesting a uniform particle distribution for both families of AgNPs (Figure 1). On the other hand, the zeta potential demonstrated negative electrical charges in both sizes of nanoparticles. The small and larger nanoparticles proved to be more electrically stable, avoiding the risk of particle agglomeration (−35.0 ± 3.3 and −52.6 ± 8.5 mV, respectively). These results indicate that both AgNP samples presented adequate and well-defined sizes with electrical charges in their surfaces preventing particle agglomeration.

### 3.2. General Characteristics of Patients 

The general sociodemographic characteristics of subjects with and without MID are described in Table 2. Thirty-seven oral biofilms were obtained from young adult subjects (19.5 ± 4.0 years old). The more frequent disorder was DS (13 subjects) followed by ID (2 subjects) and ID/CP (2 subjects). The age of patients with MID was statistically younger (15.5–16.6 years old) compared with subjects with no disabilities (22.2 years old), where women patients were older (20.9 ± 2.9 years old) than men (18.6 ± 4.5 years old). Notably, the patients with SD were slightly older (16.6 ± 3.0 years old) compared with the ID (16.0 ± 2.8 years old) and ID/CP (15.5 ± 7.7 years old) subjects, while the patients with no disorder were the oldest (22.2 ± 2.4 years-old). Also, women patients presented a higher age (17.5 ± 1.9–22.7 ± 1.7 years old) than men (10.0 ± 0.0–21.8 ± 2.8 years old) for all groups. On the other hand, the frequency of men was commonly higher (50.0–69.2%) compared with women patients (30.8–50.0%) for all groups; however, no statistical differences among study groups with and without MID were identified (*p* > 0.05). These results suggest that gender was uniformly distributed for all study groups, but age tended to be greater in subjects with no disorder. 

### 3.3. Initial Bacterial Growth 

**Figure 2:**
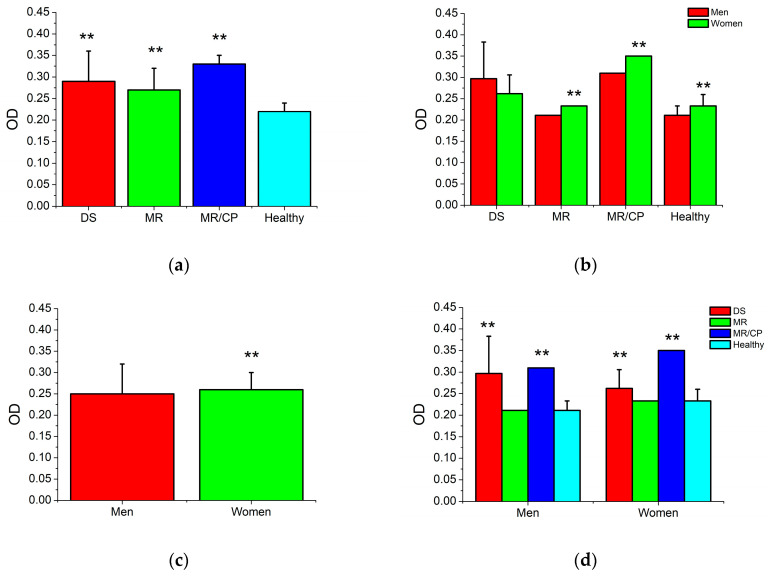
Initial bacterial growth of oral biofilms from patients with and without disabilities. DS = Down syndrome, ID = intellectual disorder, ID/CP = intellectual disorder with cerebral palsy. Optical density results are measured in absorbance units (a.u.) and expressed in mean and standard deviation. Two asterisks indicate statistical differences respecting healthy and male subjects, respectively (*p* < 0.01). (**a**) Bacterial growth rate according to the presence and type of disability. (**b**,**d**) Bacterial growth rate according to the presence and type of disability and gender. (**c**) Bacterial growth rate according to gender.

Figure 2 shows the results of the initial bacterial growth of oral biofilms associated with the different MIDs. The initial growth of the microorganisms involved in the different oral biofilms of the MID presented variations in bacterial cell proliferation. Oral biofilms taken from subjects with ID and CP showed significantly higher levels of bacterial growth (0.33 ± 0.02 a.u.) followed by oral biofilms from DS (0.29 ± 0.07 a.u.) and ID (0.27 ± 0.05 a.u.) patients. Note that biofilms from subjects with no disabilities showed the statistically lowest bacterial growth rate (0.22 ± 0.03 a.u.) (Figure 2a,d). Regarding gender, bacterial growth identified women as possessing the type of oral biofilms that had a statistically higher predisposition (0.026 ± 0.04 a.u.) according to bacterial cell proliferation compared with men (0.025 ± 0.07 a.u.) (Figure 2c). This result included oral biofilms from women with ID, ID/CP, and healthy subjects (Figure 2b). Therefore, the bacterial growth rate is highly related to the type of oral biofilm and gender, particularly those obtained from patients with MID and female patients.

### 3.4. Bactericidal Activity of AgNPs in Oral Biofilms

**Figure 3:**
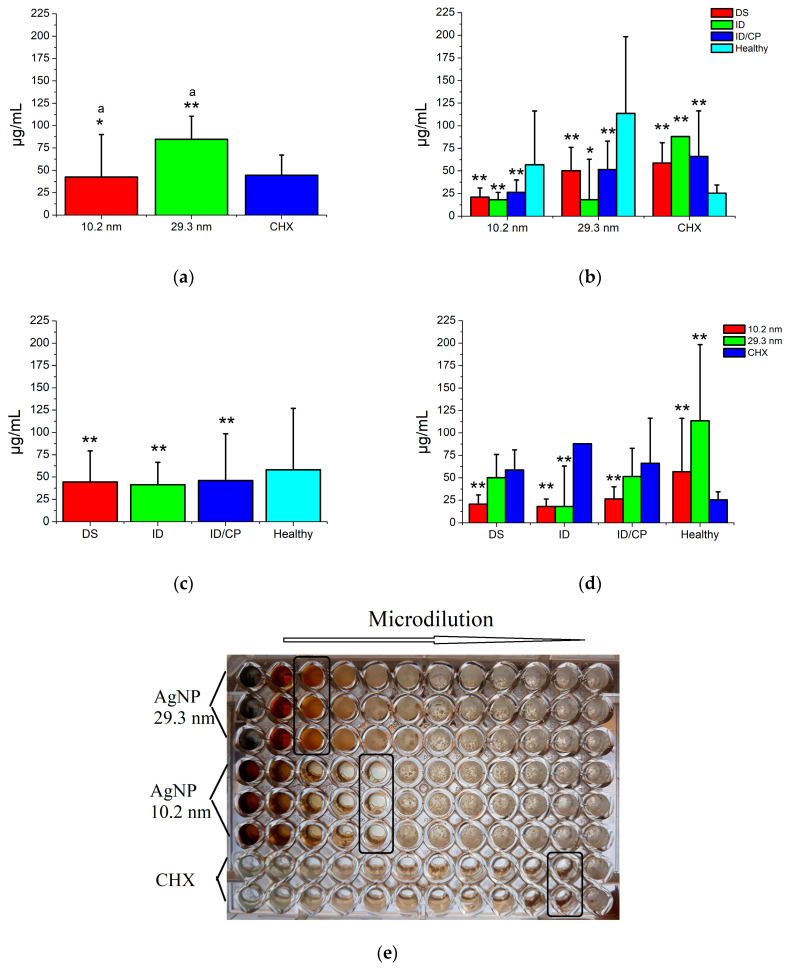
Bactericidal activity of AgNPs against oral biofilms from patients with and without disabilities. DS = Down syndrome, ID = intellectual disorder, ID/CP = intellectual disorder with cerebral palsy. One asterisk indicates *p* < 0.05; two asterisks indicate *p* < 0.01. The independent comparisons were made regarding healthy subjects and the CHX solution (control group), respectively. Similar letters indicate statistical differences between antimicrobial solutions. (**a**) Antimicrobial activity of AgNP and CHX solutions; (**b**) antimicrobial activity of AgNP and CHX solutions according to the presence and type of disability; (**c**) antimicrobial sensibility of subjects with and without disability (average of MIC from all antimicrobials); (**d**) antimicrobial sensibility of subjects with and without disability according to both families of AgNP and CHX solutions; and (**e**) representative microdilution plate of oral biofilm from a healthy subject (black squares indicate MIC).

Figure 3 shows the antimicrobial activity of AgNPs against oral biofilms associated with and without MID. In general, both kinds of AgNPs had good bacterial inhibition activity for all oral biofilms taken from patients with and without disorders; however, the smaller Ag particles were significantly more effective (42.5 ± 47.3 µg/mL) than the larger ones (84.6 ± 25.9 µg/mL), even for the CHX solution (44.5 ± 22.5 µg/mL) (Figure 3a). In addition, the more resistant microorganisms to the action of antimicrobials were statistically presented by oral biofilms from subjects with no disabilities (58.0 ± 68.8 µg/mL), followed by patients with ID/CP (45.9 ± 52.5 µg/mL) and DS (44.3 ± 35.1 µg/mL), and the most sensitive bacteria were observed in biofilms from ID patients (41.2 ± 25.2 µg/mL) (Figure 3b,c). Interestingly, the bactericidal activity of both types of AgNPs acted similarly for all oral biofilms, even for patients with no disability (healthy subjects). Therefore, the bacterial growth inhibition of the CHX solution in patients with MID had statistically lower efficacy (58.5–87.8 µg/mL) compared with the AgNP solutions (18.1–26.3 and 18.1–51.4 µg/mL, respectively) (Figure 3b,d). Although the CHX solution showed better bactericidal efficacy on oral biofilms from healthy patients (25.3 ± 9.0 µg/mL) than AgNPs (56.7 ± 59.4 and 113.6 ± 84.5 µg/mL, respectively) (Figure 3e), both families of AgNPs had higher and significant bacterial growth inhibition efficiency against biofilms from disabled patients (18.1–51.4 µg/mL) compared with the CHX solution (58.5–87.8 µg/mL) (Figure 3d). This means AgNPs have good bactericidal efficiency for all oral biofilms taken from patients with and without MID. Moreover, better antimicrobial associations were related to the type of antimicrobial treatment, the particle size of AgNPs, as well as the type of disability, in which the smaller nanoparticles acted much better to inhibit the bacterial growth in patients with disorders, while the CHX solution was the best antimicrobial treatment for healthy patients.

**Figure 4:**
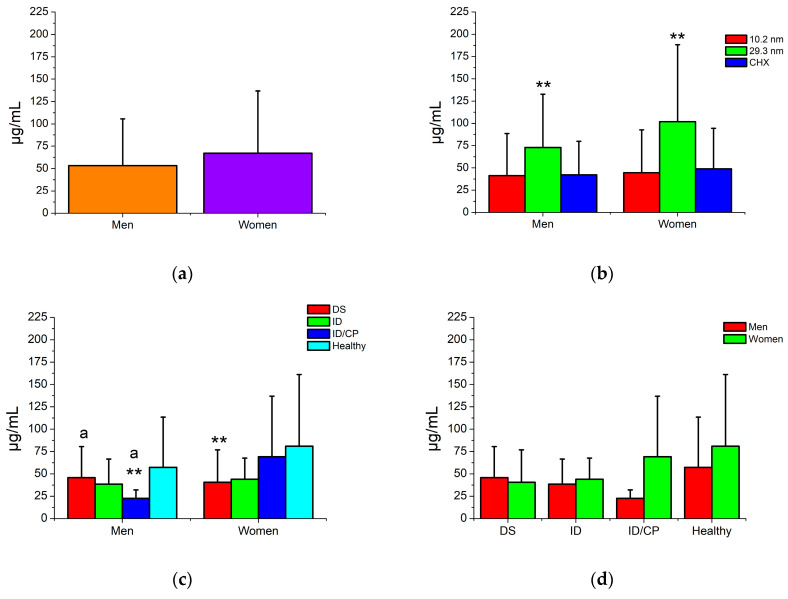
Bactericidal activity of AgNPs against oral biofilms from patients with and without disabilities according to gender. DS = Down syndrome, ID = intellectual disorder, ID/CP = intellectual disorder with cerebral palsy. Two asterisks indicate *p* < 0.01. Independent comparisons were made regarding healthy subjects and the CHX solution (control group), respectively. Similar letters indicate statistical differences between types of disability. (**a**) Antimicrobial sensibility to AgNP and CHX solutions (average of both AgNP samples and CHX). (**b**) Antimicrobial sensibility according to the type of AgNP and CHX solutions. (**c**,**d**) Antimicrobial sensibility according to the presence and type of disability (average of both AgNP samples and CHX according to gender).

Figure 4 shows the bactericidal activity of AgNPs on oral biofilms from patients with and without MID according to gender. In general, female patients presented a tendency to have microorganisms that are more resistant in their biofilms to the action of antimicrobial therapies (67.1 ± 69.6 µg/mL) compared with males (53.3 ± 52.3 µg/mL), even for any type of disability; however, no significant differences were found (Figure 4a,d). Furthermore, the smaller AgNPs and CHX solution had statistically similar antimicrobial activity for men (41.2–42.1 µg/mL) and women subjects (44.7–48.8 µg/mL) compared with larger nanoparticles (72.9 and 101.8 µg/mL, respectively) (Figure 4b). Healthy patients, mainly females, demonstrated higher resistant activity to the antimicrobials (57.2-81.1 µg/mL) than those microorganisms involved in biofilms from patients with any type of MID (22.6–69.2 µg/mL) (Figure 4c). In addition, specific oral biofilms had notable variations in their resistance level to antimicrobial treatments among the genders (Figure 4c). Thus, the male patients with DS showed the most resistant microorganism (45.8 ± 34.7 µg/mL), followed by ID (38.6 ± 28.0 µg/mL), and, on the other hand, the most sensitive bacteria were found in male subjects with ID with CP (22.6 ± 9.5 µg/mL). Contrastingly, the women subjects with ID and CP had the most increased resistance levels (69.2 ± 67.7 µg/mL), while the most sensitive biofilms were from men patients with ID (43.9 ± 23.7 µg/mL) and DS conditions (40.7 ± 36.1 µg/mL). These results suggest that gender, predominantly female patients, tends to have high levels of bacterial resistance; but, at the same time, specific genders are associated with particular levels of bacterial resistance from different oral biofilms, principally in patients with MID.

**Figure 5:**
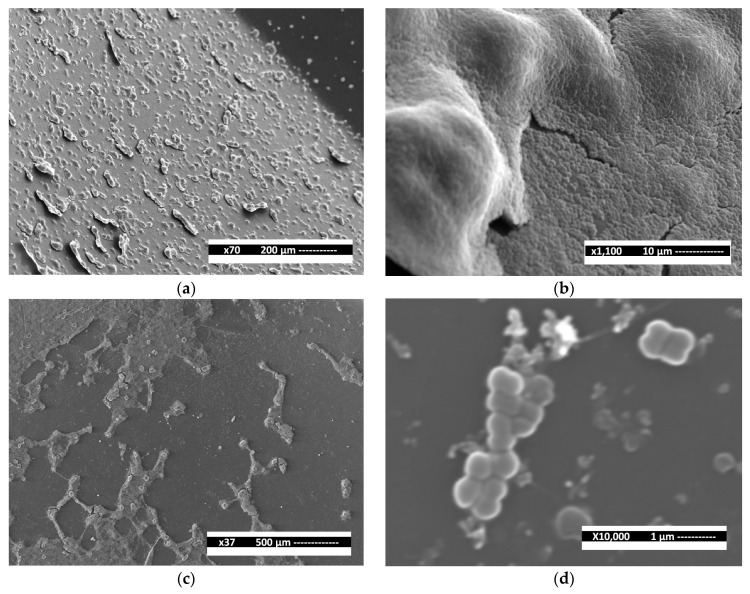
SEM micrographs of *S. mutans* exposed to smaller AgNPs (10.2 nm). (**a**,**b**) Bacteria with no antimicrobial treatment. (**c**,**d**) Bacteria exposed to AgNPs.

Figure 5 shows SEM micrographs of the bactericidal and antibiofilm activity of AgNPs in a reference strain of *S. mutans*. Figure 5a,b present agglomerated and well-adhered bacterial cells, conforming to a regular structure of oral biofilm with good relationships between cell–cell and cell–surface. The thickness of biofilm was approximately observed from 3 to 15 µm (Figure 5b). On the other hand, bacterial cells exposed to AgNPs (10.2 nm) showed growth inhibition zones with adherence alterations in the structure of the biofilm, limiting the adherence relationships between cell–cell and cell–surface (Figure 5c,d).

Table 3 presents Spearman’s rho correlations of OD and MIC values for AgNPs according to age from MID and healthy patients. Some significant correlations in the initial bacterial growth with the age of some study groups were identified. The initial bacterial growth rate from DS and healthy subjects showed positive and significant correlations with age (*rho* = 0.367, *p* = 0.000, and *rho* = 0.965, *p* = 0.000, respectively). On the other hand, the nanoparticle content for both sizes of AgNPs demonstrated positive and significant correlations for all groups with and without MID (AgNP 10.2 nm *rho* = 0.285, *p* = 0.002; and AgNP 29.3 nm, *rho* = 0.443, *p* = 0.000). Remarkably, the smaller AgNPs had positive and negative significant correlations for patients with ID (*rho* = 0.905, *p* = 0.013) and healthy subjects (*rho* = −0.322, *p* = 0.012), respectively. Finally, the patients with some type of MID tended to have more positive correlations with the concentrations of both types of AgNPs (*rho* = 0.144 to 0.905) compared to healthy subjects (*rho* = −0.011 to −0.322). These results indicate that the bacterial growth rate in patients with DS and healthy subjects increased gradually with age. Furthermore, the concentration of both AgNP families in patients with MID had gradually increasing tendencies with age, while the nanoparticle content for healthy patients diminished when age increased.

### 3.5. Identification and Distribution of Bacterial Species by PCR 

Table 4 shows the presence and frequency of bacterial species identified using PCR. In general, the oral biofilms used for this molecular assay were from young subjects (15.7 ± 0.7 years old), even for patients with (15.7 ± 0.7 years old) and without (15.7 ± 0.9 years old) disabilities with homogeneous distribution according to gender for both groups (50% for males and 50% for females). The bacterial profile indicated that *S. mutans* was the most prevalent bacteria (100%) for all groups, followed by *P. intermedia* (91.6%), *T. forsythia* (75.0%), *T. denticola* (75.0%), *P. gingivalis* (66.6%), *F. nucleatum* (66.6%), *S. sobrinus* (50.0%), and, finally, *A. actinomycetemcomitans*, which was the least frequent (8.3%). Therefore, the frequencies of *S. sobrinus* (62.5%), *P. gingivalis* (87.5%), *P. intermedia* (100%), and *A. actinomycetemcomitans* (12.5%) were higher in patients with disabilities compared with those with no disabilities (25.0, 25.0, 75.0, and 0.0%, respectively), while *S. mutans*, *T. forsythia*, and *T. denticola* had similar distribution between groups. Mainly, DS patients showed higher distributions for *S. sobrinus* (50.0%), *P. gingivalis* (83.3%), *T. forsythia* (83.3%), *T. denticola* (83.3%), *P. intermedia* (100%), *F. nucleatum* (83.3%), and *A. actinomycetemcomitans* (16.6%) than patients without disabilities (0.0–75%). In addition, bacterial profiles from subjects with disabilities had more presence of bacterial (71.8%) than patients without disabilities (56.2%); however, the DS patients had the highest bacterial distribution (74.9%). These results suggest that oral biofilms have specific and different bacterial profiles between subjects with disabilities and without disabilities, predominately for DS subjects. Although in some particular cases, patients with ID and ID/CP conditions had higher distributions of specific species (*S. sobrinus*, *P. gingivalis*, *T. forsythia*, and *T. denticola*) in comparison with DS and healthy patients, the insufficient number of cases for ID and ID/CP groups limit clinical and microbiological interpretation.

## 4. Discussion

This study determined that both types of AgNPs had good bacterial growth inhibition activity against microorganisms found in oral biofilms isolated from patients with and without MID. Likewise, the best antimicrobial action of AgNPs was highly related to smaller nanoparticles for all bacterial samples, principally in oral biofilms from subjects with MID. On the other hand, the most resistant bacteria to all antimicrobial solutions, including AgNPs, were in oral biofilms from healthy patients (with no MID). In contrast, the most sensitive bacteria were in oral biofilms from subjects with ID, DS, and ID/CP, respectively. Although there were no significant differences in the bactericidal activity of AgNPs according to gender, women patients presented a greater trend in antimicrobial resistance than men for any type of condition. In addition, the bacterial growth rate showed the significantly highest levels in oral biofilms from patients with MID, predominantly in subjects with ID/CP, followed by DS and ID, and, finally, the lowest bacterial growth was seen in biofilms from healthy patients. Finally, the significant correlations determined that the bacterial growth rate and the content of AgNPs increased gradually with the age of patients and, even, for some specific groups such as DS patients. However, negative and significant correlations were also identified in women subjects and the healthy group correlating similar variables. To our knowledge, this is the first study that evaluated the antimicrobial activity of AgNPs with two particle sizes against different types of oral biofilms taken from subjects with and without MID. These results provide a better understanding of the bactericidal mechanisms of AgNPs exposed to microorganisms involved in oral biofilms with more realistic microbiological conditions, in which the bacterial growth inhibition ability of AgNPs and their physical and chemical interactions with the clinical oral biofilms could improve and preserve the oral health of patients with MID. 

Various studies have evaluated the antimicrobial effect of AgNPs against multiple species and their relationship with their physicochemical properties, which can be modified by factors such as chemical and electrical stability, particle dispersion, size, and other physical and chemical properties [39,40]. According to this study, AgNPs were adjusted to reach an alkaline pH with sodium (pH 11) and ammonium (pH 10) hydroxides, obtaining narrow and well-distributed particle sizes (10.2 ± 0.7 and 29.3 ± 2.3 nm) with spherical shape (Table 1 and Figure 1). Also, our results suggest that AgNPs had adequate electrical charges for the smaller AgNPs (−35.0 ± 3.3 mV), while the larger ones still promoted more stable nanoparticles (−52.6 ± 8.5 mV), limiting their agglomeration [41]. 

It is worth noting that the antimicrobial and antibiofilm activity of AgNPs has been widely studied for a great variety of microorganisms, including species related to the oral cavity [42,43,44,45,46]; however, few studies in the literature have determined the bactericidal level of AgNPs against oral biofilms isolated from patients. One study evaluated the antimicrobial activity of two sizes of AgNPs, in which oral biofilms related to young adult patients were investigated [47]. These authors reported that AgNPs showed good bactericidal properties for all oral biofilms, significantly limiting the bacterial growth of oral biofilms associated with dental caries and periodontal disease. Also, these works suggested that the mechanism of action of AgNPs could be related to the physiochemical properties of AgNPs and also to sociodemographic and microbiological factors such as gender, age, habits, type and distribution of biofilms, type of bacterial cell wall, and others [47]. Similarly, other authors have also investigated the bactericidal activity of AgNPs against a wide sampling of oral biofilms from patients associated with dental caries and periodontal disease [26,48]. In these studies, different and well-distributed sizes of AgNPs (5.2–5.4 and 17.5–37.4 nm) with negative zeta potential (from −32.6 to −52.6 mV) were used. These authors concluded that both sizes of AgNPs exerted good bactericidal properties on all types of oral biofilms and intervened in the control and progression of dental caries and periodontal disease [26,48]. On the other hand, studies have investigated the antimicrobial activity of AgNPs against biofilms and clinically isolated bacterial strains from biofilms using other types of microorganisms [27,29], such as *Candida albicans* strains [29] and strains isolated from denture stomatitis lesions [27]. Another research on AgNPs prepared by laser ablation and exposed to multispecies biofilms (*Staphylococcus aureus* or oral mixed bacterial flora composed of *Streptococcus oralis*, *Actinomyces naeslundii*, *Veionella dispar*, and *Porphyromonas gingivalis*) related to peri-implantitis of oral implants reported that AgNPs induced a higher biofilm inhibition, acting as a promising antimicrobial alternative to control dental implant infections [49]. Our results suggest that AgNPs present adequate bactericidal and antibiofilm efficacy against all biofilms tested from patients with and without MID. In contrast, nanoparticles with the smallest size exhibited the best antimicrobial effect, while the biofilms with the most sensitive biofilms were related to patients with some type of MID (Figure 3). Moreover, biofilms from female patients had more increased levels of antimicrobial resistance to AgNPs than male patients. However, no statistical differences were found, suggesting an interesting predisposition and relationship with gender, even for the different types of biofilms (with and without disorders) (Figure 4). Additionally, the correlation results showed that the bacterial growth rate of DS and healthy patients was age-dependent. Regarding nanoparticle content, both AgNP samples and age had similar and significant tendencies for patients with disabilities, while nanoparticle content and age showed an opposite correlation for healthy patients, suggesting that the content of AgNPs related to the age of patients depends on the type of biofilm (Table 3). These results suggest that AgNPs’ antimicrobial and antibiofilm activity is strongly related to physical properties, such as a small size and electrical surface charge. A possible mechanism is that chemical affinities of AgNPs and silver ions with thiol, amino, and hydroxyl groups of bacterial cells may bind to the cell membrane, penetrating drastically and affecting the permeability of the cell, respiration process, activation of proteins, and leading to death [50,51]. In parallel, AgNPs could interfere with the production of extracellular polysaccharides, such as GTF and FTF enzymes, leading to no-adhesion activity between cell–cell and cell–surface and, finally, a weak structure of biofilms [45]. Nevertheless, at the same time, a mixed antimicrobial mechanism involving microbiological, clinical, systemic, and sociodemographic conditions, such as biofilm, disorder, age, and gender, could play an important role in AgNP bactericidal activity. 

It is well known that antibiotic resistance is a constantly evolving global problem [10]. However, there are specific oral bacteria that have enhanced tolerance or, even, resistance to a wide spectrum of antimicrobials used commonly in dentistry, many of which are considered gold antimicrobial standards [11,12]. Authors have identified some age groups in the range from 26 to 50 years old and the female gender with the highest multi-resistant levels [12] with more periodontal problems than men [52,53,54]; however, exclusive oral bacteria related to periodontal disease and dental caries also promote bacterial resistance according to particular biofilms [55]. Our results indicated that female subjects presented increased bacterial growth rates and needed significantly higher amounts of AgNPs, or even the CHX solution, compared with males (*p* < 0.05). A tentative explanation is that women have particular physiological and metabolic stages that alter the hormonal conditions, such as puberty, menstrual periods, menopause, the use of contraceptives, or, even pregnancy, which generate a modified immune response, promoting the occurrence of oral diseases [54,56]. 

Studies have focused on understanding the bacteria distribution and clinical predispositions related to specific oral bacteria from patients with various types of disabilities. Authors have concluded that DS patients have demonstrated the most critical clinical and periodontal parameters and different microbiota compositions related to periodontal bacteria in comparison with subjects affected by other MIDs [16,17,57]. The above suggests that the action mechanism of AgNPs, although it remains unclear, not only depends on the physicochemical properties of the nanoparticles and external and internal interactions with the bacterial cells [50,58], but also on microbiological and clinical peculiarities from specific oral biofilms that include the type and number of bacterial distribution, types of oral and systemic diseases, immunological, metabolic, and hormonal conditions, age, gender, and, probably, other relevant factors such as lifestyle and habits [26]. 

Similarly, this study recommends the use of AgNPs as a potential antimicrobial agent to control the growth proliferation of oral biofilms related to patients with particular MIDs, particularly in subjects with DS, ID, and ID/CP, who are highly affected by oral pathogens etiologically involved in dental caries and periodontal disease. Although some investigations have suggested the safe use of these metallic nanostructured materials in biomedical applications [59,60], other studies have reported the potential toxicological damage of AgNPs when they interact with particular organs or cells [22,61]. Of course, many other scientific protocols for these nanomaterials should be conducted to elucidate the toxicological mechanism, better administration routes, and more adequate doses to create more predictable and safer therapeutics for biomedical applications. Probably, the AgNPs incorporated into mouthwashes, toothpaste, varnishes, toothpicks, toothbrushes, and other regular dental instruments for oral hygiene could be used as complementary antimicrobial tools in the preservation of oral health during conventional procedures of oral maintenance in patients with MID. One of the most important limitations of this work, among others, was the sampling size, which was relatively narrow with only seventeen patients with MID and twenty subjects without MID (controls). Although there is no scientific information that includes this number of patients, it is necessary to examine other scientific protocols using AgNPs with more extensive sampling, different oral biofilms, the inclusion of other relevant sociodemographic, clinical, and oral conditions, more realistic simulated conditions, or even, other nanostructured materials. This will certainly create a better understanding of the behavior of these metallic nanoparticles as an alternative and complementary instrument during conventional oral hygiene, leading to the safe use of AgNPs for the control and prevention of oral biofilms in humans with special requirements. 

## 5. Conclusions

AgNPs exhibited adequate bacterial growth inhibition against all oral biofilms related to patients with MID. The main associations among the antimicrobial activity of AgNPs were indicated in the particle size and type of oral biofilm. It was determined that the smaller size was statistically the most effective, in contrast, the oral biofilms from patients with disabilities, particularly ID subjects, were the most sensitive biofilms to the action of AgNPs. Notably, gender had no significant differences in the bactericidal activity of AgNPs; however, an interesting tendency was found in female subjects as they presented more antimicrobial resistance than males. According to correlations with age, the biofilms from patients with disabilities need a gradual increase in the number of nanoparticles with respect to age, while the biofilms from patients without disabilities showed an opposite correlation behavior. Furthermore, the content of both AgNP families in patients with MID had gradually increasing tendencies with age, while the nanoparticle content in healthy patients diminished when age increased. In general, the clinical significance of AgNPs can be represented by a latent and effective alternative for the control and prevention of the most frequent oral biofilms, principally related to dental caries and periodontal disease, in patients with MID. Consequently, new and novel scientific approaches based on AgNPs that include other sociodemographic factors, as well as medical and oral conditions, e.g., age, gender, diet, race, current medications, habits, previous medical and dental procedures, etc., should be implemented.

## Figures and Tables

**Table 1:** Characterization of AgNPs.

AgNP (nm)	DLS (nm)	Shape	Initial Concentration (µg/mL)	Zeta Potential (mV)
First preparation (small)	10.2 ± 0.7	Spherical	1070	−35.0 ± 3.3
Second preparation (large)	29.3 ± 2.3	Spherical	1070	−52.6 ± 8.5

DLS = dynamic light scattering. DLS and zeta potential results are expressed as mean, standard deviation, and zeta deviation, respectively.

**Table 2:** General sociodemographic distribution of the study groups.

Variable	Motor and Intellectual Disabilities *n* = 17 Subjects			Control (Healthy) *n* = 20 Subjects	Total *n* = 37 Subjects	*p*-Value
	DS *n* = 13 Subjects	ID *n* = 2 Subjects	ID/CP *n* = 2 Subjects			
Mean ± SD						
Age (years old)	16.6 ± 3.0	16.0 ± 2.8	15.5 ± 7.7	22.2 ± 2.4	19.5 ± 4.0	0.000 *
Women	17.5 ± 1.9	18.0 ± 0.0	21.0 ± 0.0	22.7 ± 1.7	20.9 ± 2.9	
Men	16.3 ± 3.4	14.0 ± 0.0	10.0 ± 0.0	21.8 ± 2.8	18.6 ± 4.5	
Frequency (%)						
Gender	13 (35.1)	2 (5.4)	2 (5.4)	20 (54.1)	37 (100)	0.843
Women	4 (30.8)	1 (50.0)	1 (50.0)	9 (45.0)	15 (40.5)	
Men	9 (69.2)	1 (50.0)	1 (50.0)	11 (55.0)	22 (59.5)	

Patient age is expressed in mean and standard deviation. DS = Down syndrome; ID = intellectual disorder; ID/CP = intellectual disorder with cerebral palsy. The student’s *t*-test and Pearson´s chi-square analysis were used to determine statistical differences in age and gender between groups, respectively. The asterisk symbol (*) indicates statistical differences between study groups (*p* < 0.05).

**Table 3:** Spearman’s rho correlation of OD and MIC values for AgNPs according to age from MID and healthy patients.

Variable	MID (*n* = 17 Subjects)						Healthy *n* = 20 Subjects (*rho*)	*p*-Value	Total *n* = 37 Subjects (*rho*)	*p*-Value
	DS *n* = 13 Subjects (*rho*)	*p*-Value	ID *n* = 2 Subjects (*rho*)	*p*-Value	ID/CP *n* = 2 Subjects (*rho*)	*p*-Value				
	**Age**									
OD	0.367	0.000 **	1.00	-	1.00	-	0.965	0.000 **	−0.070	0.232
AgNP 10.2 nm	0.304	0.060	0.905	0.013 **	0.577	0.230	−0.322	0.012 *	0.285	0.002 **
AgNP 29.3 nm	0.144	0.382	0.000	1.00	0.000	1.00	−0.011	0.933	0.443	0.000 **

DS = Down syndrome; ID = intellectual disorder; ID/CP = intellectual disorder with cerebral palsy; OD = optical density. * indicates *p* < 0.05; ** indicates *p* < 0.01. - Not applicable.

**Table 4:** Presence and distribution of bacterial species involved in oral biofilms detected by PCR.

Variable	MID *n* = 8 Subjects (%)				Control (Healthy) *n* = 4 Subjects (%)	Total *n* = 12 Subjects (%)
	DS *n* = 6 Subjects (%)	ID *n* = 1 Subject (%)	ID/CP *n* = 1 Subject (%)	Total of Disabilities *n* = 8 Subjects (%)		
Age (years)	15.8 ± 0.7	15.0	16.0	15.7 ± 0.7	15.7 ± 0.9	15.7 ± 0.7
Women	15.6 ± 0.5	15.0	-	15.5 ± 0.5	15.5 ± 0.7	15.5 ± 0.5
Men	16.0 ± 1.0	-	16.0	16.0 ± 0.8	16.0 ± 1.4	16.0 ± 0.8
Gender						
Women	3 (50.0)	1 (100)	-	4 (50.0)	2 (50.0)	6 (50.0)
Men	3 (50.0)	-	1 (100)	4 (50.0)	2 (50.0)	6 (50.0)
Bacterial strains						
*S. mutans*	6 (100)	1 (100)	1 (100)	8 (100)	4 (100)	12 (100)
*S. sobrinus*	3 (50.0)	1 (100)	1 (100)	5 (62.5)	1 (25.0)	6 (50.0)
*P. gingivalis*	5 (83.3)	1 (100)	1 (100)	7 (87.5)	1 (25.0)	8 (66.6)
*T. forsythia*	5 (83.3)	0 (0.0)	1 (100)	6 (75.0)	3 (75.0)	9 (75.0)
*T. denticola*	5 (83.3)	1 (100)	0 (0.0)	6 (75.0)	3 (75.0)	9 (75.0)
*P. intermedia*	6 (100)	1 (100)	1 (100)	8 (100)	3 (75.0)	11 (91.6)
*F. nucleatum*	5 (83.3)	0 (0.0)	0 (0.0)	5 (62.5)	3 (75.0)	8 (66.6)
*A. actinomycetemcomitans*	1 (16.6)	0 (0.0)	0 (0.0)	1 (12.5)	0 (0.0)	1 (8.3)

Values for age are expressed as mean and standard deviation. PCR = polymerase chain reaction; DS = Down syndrome; ID = intellectual disorder; ID/CP = intellectual disorder with cerebral palsy. - Not applicable.

## Data Availability

All data obtained during this study can be found in the research archives of the Master’s Program in Dental Sciences of the Autonomous University of Ciudad Juarez and can be requested from the corresponding author.

## References

[1] World Health Organization Disability. https://www.who.int/news-room/fact-sheets/detail/disability-and-health.

[2] Pan American Health Organization Disability. https://www.paho.org/en/topics/disability.

[3] World Health Organization Global Oral Health Status Report: Towards Universal Health Coverage for Oral Health by 2030. https://www.who.int/publications/i/item/9789240061484.

[4] Wilson N.J., Lin Z., Villarosa A., Lewis P., Philip P., Sumar B., George A. (2019). Countering the Poor Oral Health of People with Intellectual and Developmental Disability: A Scoping Literature Review. BMC Public Health.

[5] Wilson N.J., Patterson-Norrie T., Villarosa A., Calache H., Slack-Smith L., Kezhekkekara S.G., George A. (2024). Supporting the Oral Health of People with Intellectual Disability: A Survey of Disability Staffs’ Knowledge, Perceptions, Disability Service Barriers, and Training. Disabil. Health J..

[6] Wilson N.J., Lin Z., Villarosa A., George A. (2019). Oral Health Status and Reported Oral Health Problems in People with Intellectual Disability: A Literature Review. J. Intellect. Dev. Disabil..

[7] Dembowska E., Jaroń A., Gabrysz-Trybek E., Bladowska J., Trybek G. (2022). Evaluation of Common Factors of Periodontitis and Cardiovascular Disease in Patients with the Acute Coronary Syndrome. Int. J. Environ. Res. Public Health.

[8] Dieguez-Perez M., de Nova-Garcia M., Mourelle-Martinez M., Bartolome-Villar B. (2016). Oral Health in Children with Physical (Cerebral Palsy) and Intellectual (Down Syndrome) Disabilities: Systematic Review I. J. Clin. Exp. Dent..

[9] van Duin D., Paterson D.L. (2020). Multidrug-Resistant Bacteria in the Community. Infect. Dis. Clin. N. Am..

[10] Roth M., Goerke P., Holtmann C., Frings A., MacKenzie C.R., Geerling G. (2022). Spectrum and Resistance in Bacterial Infections of the Ocular Surface in a German Tertiary Referral Center 2009–2019. Graefe’s Arch. Clin. Exp. Ophthalmol..

[11] Cieplik F., Jakubovics N.S., Buchalla W., Maisch T., Hellwig E., Al-Ahmad A. (2019). Resistance toward Chlorhexidine in Oral Bacteria—Is There Cause for Concern?. Front. Microbiol..

[12] Loyola-Rodriguez J.P., Ponce-Diaz M.E., Loyola-Leyva A., Garcia-Cortes J.O., Medina-Solis C.E., Contreras-Ramire A.A., Serena-Gomez E. (2018). Determination and Identification of Antibiotic-Resistant Oral Streptococci Isolated from Active Dental Infections in Adults. Acta Odontol. Scand..

[13] Lamont R.J., Koo H., Hajishengallis G. (2018). The Oral Microbiota: Dynamic Communities and Host Interactions. Nat. Rev. Microbiol..

[14] Espinosa-Cristóbal L.F., López-Ruiz N., Cabada-Tarín D., Reyes-López S.Y., Zaragoza-Contreras A., Constandse-Cortéz D., Donohué-Cornejo A., Tovar-Carrillo K., Cuevas-González J.C., Kobayashi T. (2018). Antiadherence and Antimicrobial Properties of Silver Nanoparticles against Streptococcus Mutans on Brackets and Wires Used for Orthodontic Treatments. J. Nanomater..

[15] Loyola-Rodriguez J.P., Martinez-Martinez R.E., Flores-Ferreyra B.I., Patiño-Marin N., Alpuche-Solis A.G., Reyes-Macias J.F. (2007). Distribution of Streptococcus Mutans and Streptococcus Sobrinus in Saliva of Mexican Preschool Caries-Free and Caries-Active Children by Microbial and Molecular (PCR) Assays. J. Clin. Pediatr. Dent..

[16] Khocht A., Yaskell T., Janal M., Turner B.F., Rams T.E., Haffajee A.D., Socransky S.S. (2012). Subgingival Microbiota in Adult Down Syndrome Periodontitis. J. Periodontal Res..

[17] Contaldo M., Lucchese A., Romano A., Della Vella F., Di Stasio D., Serpico R., Petruzzi M. (2021). Oral Microbiota Features in Subjects with Down Syndrome and Periodontal Diseases: A Systematic Review. Int. J. Mol. Sci..

[18] Tanaka M.H., Rodrigues T.O., Finoti L.S., Teixeira S.R.L., Mayer M.P.A., Scarel-Caminaga R.M., Giro E.M.A. (2015). The Effect of Conventional Mechanical Periodontal Treatment on Red Complex Microorganisms and Clinical Parameters in Down Syndrome Periodontitis Patients: A Pilot Study. Eur. J. Clin. Microbiol. Infect. Dis..

[19] Poppolo Deus F., Ouanounou A. (2022). Chlorhexidine in Dentistry: Pharmacology, Uses, and Adverse Effects. Int. Dent. J..

[20] Kozelskaya A.I., Verzunova K.N., Akimchenko I.O., Frueh J., Petrov V.I., Slepchenko G.B., Bakina O.V., Lerner M.I., Brizhan L.K., Davydov D.V. (2023). Antibacterial Calcium Phosphate Coatings for Biomedical Applications Fabricated via Micro-Arc Oxidation. Biomimetics.

[21] Villapún V.M., Esat F., Bull S., Dover L.G., González S. (2017). Tuning the Mechanical and Antimicrobial Performance of a Cu-Based Metallic Glass Composite through Cooling Rate Control and Annealing. Materials.

[22] Xu L., Wang Y.-Y., Huang J., Chen C.-Y., Wang Z.-X., Xie H. (2020). Silver Nanoparticles: Synthesis, Medical Applications and Biosafety. Theranostics.

[23] Seo M., Oh T., Bae S. (2021). Antibiofilm Activity of Silver Nanoparticles against Biofilm Forming Staphylococcus Pseudintermedius Isolated from Dogs with Otitis Externa. Vet. Med. Sci..

[24] das Neves P.B.A., Agnelli J.A.M., Kurachi C., de Souza C.W.O. (2014). Addition of Silver Nanoparticles to Composite Resin: Effect on Physical and Bactericidal Properties in Vitro. Braz. Dent. J..

[25] Ahmed O., Sibuyi N.R.S., Fadaka A.O., Madiehe A.M., Maboza E., Olivier A., Meyer M., Geerts G. (2022). Antimicrobial Effects of Gum Arabic-Silver Nanoparticles against Oral Pathogens. Bioinorg. Chem. Appl..

[26] Hernández-Venegas P.A., Martínez-Martínez R.E., Zaragoza-Contreras E.A., Domínguez-Pérez R.A., Reyes-López S.Y., Donohue-Cornejo A., Cuevas-González J.C., Molina-Frechero N., Espinosa-Cristóbal L.F. (2023). Bactericidal Activity of Silver Nanoparticles on Oral Biofilms Related to Patients with and without Periodontal Disease. J. Funct. Biomater..

[27] Fonseca M.S., Rodrigues D.M., Sokolonski A.R., Stanisic D., Tomé L.M., Góes-Neto A., Azevedo V., Meyer R., Araújo D.B., Tasic L. (2022). Activity of Fusarium oxysporum-Based Silver Nanoparticles on Candida spp. Oral Isolates. Nanomaterials.

[28] Chávez-Andrade G.M., Tanomaru-Filho M., Basso Bernardi M.I., de Toledo Leonardo R., Faria G., Guerreiro-Tanomaru J.M. (2019). Antimicrobial and Biofilm Anti-Adhesion Activities of Silver Nanoparticles and Farnesol against Endodontic Microorganisms for Possible Application in Root Canal Treatment. Arch. Oral Biol..

[29] Ribeiro L.G., Roque G.S.C., Conrado R., De Souza A.O. (2023). Antifungal Activity of Mycogenic Silver Nanoparticles on Clinical Yeasts and Phytopathogens. Antibiotics.

[30] Martinez-Martinez R.E., Abud-Mendoza C., Patiño-Marin N., Rizo-Rodríguez J.C., Little J.W., Loyola-Rodríguez J.P. (2009). Detection of Periodontal Bacterial DNA in Serum and Synovial Fluid in Refractory Rheumatoid Arthritis Patients. J. Clin. Periodontol..

[31] Martínez-Martínez R.E., Moreno-Castillo D.F., Loyola-Rodríguez J.P., Sánchez-Medrano A.G., Miguel-Hernández J.H.S., Olvera-Delgado J.H., Domínguez-Pérez R.A. (2016). Association between Periodontitis, Periodontopathogens and Preterm Birth: Is It Real?. Arch. Gynecol. Obstet..

[32] Tran S.D., Rudney J.D. (1996). Multiplex PCR Using Conserved and Species-Specific 16S RRNA Gene Primers for Simultaneous Detection of Actinobacillus Actinomycetemcomitans and Porphyromonas Gingivalis. J. Clin. Microbiol..

[33] Stubbs S., Park S.F., Bishop P.A., Lewis M.A.O. (1999). Direct Detection of Prevotella Intermedia and P. Nigrescens in Suppurative Oral Infection by Amplification of 16S RRNA Gene. J. Med. Microbiol..

[34] Watanabe K., Frommel T.O. (1996). Porphyromonas Gingivalis, Actinobacillus Actinomycetemcomitans and Treponema Denticola Detection in Oral Plaque Samples Using the Polymerase Chain Reaction. J. Clin. Periodontol..

[35] Ashimoto A., Chen C., Bakker I., Slots J. (1996). Polymerase Chain Reaction Detection of 8 Putative Periodontal Pathogens in Subgingival Plaque of Gingivitis and Advanced Periodontitis Lesions. Oral Microbiol. Immunol..

[36] Poulsen K., Ennibi O.-K., Haubek D. (2003). Improved PCR for Detection of the Highly Leukotoxic JP2 Clone of Actinobacillus Actinomycetemcomitans in Subgingival Plaque Samples. J. Clin. Microbiol..

[37] Hoshino T., Kawaguchi M., Shimizu N., Hoshino N., Ooshima T., Fujiwara T. (2004). PCR Detection and Identification of Oral Streptococci in Saliva Samples Using Gtf Genes. Diagn. Microbiol. Infect. Dis..

[38] Yoshida A., Suzuki N., Nakano Y., Kawada M., Oho T., Koga T. (2003). Development of a 5’ Nuclease-Based Real-Time PCR Assay for Quantitative Detection of Cariogenic Dental Pathogens Streptococcus Mutans and Streptococcus Sobrinus. J. Clin. Microbiol..

[39] Ullah Khan S., Saleh T.A., Wahab A., Ullah Khan M.H., Khan D., Ullah Khan W., Rahim A., Kamal S., Ullah Khan F., Fahad S. (2018). Nanosilver: New Ageless and Versatile Biomedical Therapeutic Scaffold. Int. J. Nanomed..

[40] Martínez-Castañón G.A., Niño-Martínez N., Martínez-Gutierrez F., Martínez-Mendoza J.R., Ruiz F. (2008). Synthesis and Antibacterial Activity of Silver Nanoparticles with Different Sizes. J. Nanoparticle Res..

[41] Vanitha G., Rajavel K., Boopathy G., Veeravazhuthi V., Neelamegam P. (2017). Physiochemical Charge Stabilization of Silver Nanoparticles and Its Antibacterial Applications. Chem. Phys. Lett..

[42] Pini D.d.M., Fröhlich P.C.G.R., Rigo L. (2016). Oral Health Evaluation in Special Needs Individuals. Einstein.

[43] Akbarzadeh A., Kafshdooz L., Razban Z., Dastranj Tbrizi A., Rasoulpour S., Khalilov R., Kavetskyy T., Saghfi S., Nasibova A.N., Kaamyabi S. (2018). An Overview Application of Silver Nanoparticles in Inhibition of Herpes Simplex Virus. Artif. Cells Nanomed. Biotechnol..

[44] de Lacerda Coriolano D., de Souza J.B., Bueno E.V., Medeiros S.M. (2021). de F.R. dos S.; Cavalcanti, I.D.L.; Cavalcanti, I.M.F. Antibacterial and Antibiofilm Potential of Silver Nanoparticles against Antibiotic-Sensitive and Multidrug-Resistant Pseudomonas Aeruginosa Strains. Braz. J. Microbiol..

[45] Espinosa-Cristóbal L.F., Martínez-Castañón G.A., Téllez-Déctor E.J., Niño-Martínez N., Zavala-Alonso N.V., Loyola-Rodríguez J.P. (2013). Adherence Inhibition of Streptococcus Mutans on Dental Enamel Surface Using Silver Nanoparticles. Mater. Sci. Eng. C. Mater. Biol. Appl..

[46] Nafarrate-Valdez R., Martínez-Martínez R., Zaragoza-Contreras E., Áyala-Herrera J., Domínguez-Pérez R., Reyes-López S., Donohue-Cornejo A., Cuevas-González J., Loyola-Rodríguez J., Espinosa-Cristóbal L. (2022). Anti-Adherence and Antimicrobial Activities of Silver Nanoparticles against Serotypes C and K of Streptococcus Mutans on Orthodontic Appliances. Medicina.

[47] Espinosa-Cristóbal L.F., Holguín-Meráz C., Zaragoza-Contreras E.A., Martínez-Martínez R.E., Donohue-Cornejo A., Loyola-Rodríguez J.P., Cuevas-González J.C., Reyes-López S.Y. (2019). Antimicrobial and Substantivity Properties of Silver Nanoparticles against Oral Microbiomes Clinically Isolated from Young and Young-Adult Patients. J. Nanomater..

[48] Jiménez-Ramírez A.J., Martínez-Martínez R.E., Ayala-Herrera J.L., Zaragoza-Contreras E.A., Domínguez-Pérez R.A., Reyes-López S.Y., Donohue-Cornejo A., Cuevas-González J.C., Silva-Benítez E.L., Espinosa-Cristóbal L.F. (2021). Antimicrobial Activity of Silver Nanoparticles against Clinical Biofilms from Patients with and without Dental Caries. J. Nanomater..

[49] Pérez-Tanoira R., Fernández-Arias M., Potel C., Carballo-Fernández R., Pérez-Castro S., Boutinguiza M., Górgolas M., Lusquiños F., Pou J. (2022). Silver Nanoparticles Produced by Laser Ablation and Re-Irradiation Are Effective Preventing Peri-Implantitis Multispecies Biofilm Formation. Int. J. Mol. Sci..

[50] Yin I.X., Zhang J., Zhao I.S., Mei M.L., Li Q., Chu C.H. (2020). The Antibacterial Mechanism of Silver Nanoparticles and Its Application in Dentistry. Int. J. Nanomed..

[51] Qing Y., Cheng L., Li R., Liu G., Zhang Y., Tang X., Wang J., Liu H., Qin Y. (2018). Potential Antibacterial Mechanism of Silver Nanoparticles and the Optimization of Orthopedic Implants by Advanced Modification Technologies. Int. J. Nanomed..

[52] Nakaya M., Tachibana H., Yamada K. (2006). Effect of Estrogens on the Interferon-Gamma Producing Cell Population of Mouse Splenocytes. Biosci. Biotechnol. Biochem..

[53] Bhardwaj A., Bhardwaj S. (2012). Effect of Menopause on Women′s Periodontium. J. Midlife. Health.

[54] Machtei E.E., Mahler D., Sanduri H., Peled M. (2004). The Effect of Menstrual Cycle on Periodontal Health. J. Periodontol..

[55] Loyola-Rodriguez J.P., Garcia-Cortes J.O., Martinez-Martinez R.E., Patiño-Marin N., Martinez-Castañon G.A., Zavala-Alonso N.V., Amano A. (2014). Molecular Identification and Antibiotic Resistant Bacteria Isolated from Primary Dentition Infections. Aust. Dent. J..

[56] Lipsky M.S., Su S., Crespo C.J., Hung M. (2021). Men and Oral Health: A Review of Sex and Gender Differences. Am. J. Mens. Health.

[57] Mitsuhata C., Kado N., Hamada M., Nomura R., Kozai K. (2022). Characterization of the Unique Oral Microbiome of Children with Down Syndrome. Sci. Rep..

[58] Martínez-Robles Á., Loyola-Rodríguez J., Zavala-Alonso N., Martinez-Martinez R., Ruiz F., Lara-Castro R., Donohué-Cornejo A., Reyes-López S., Espinosa-Cristóbal L. (2016). Antimicrobial Properties of Biofunctionalized Silver Nanoparticles on Clinical Isolates of Streptococcus Mutans and Its Serotypes. Nanomaterials.

[59] Munger M.A., Radwanski P., Hadlock G.C., Stoddard G., Shaaban A., Falconer J., Grainger D.W., Deering-Rice C.E. (2014). In Vivo Human Time-Exposure Study of Orally Dosed Commercial Silver Nanoparticles. Nanomedicine.

[60] Hebeish A., El-Rafie M.H., EL-Sheikh M.A., Seleem A.A., El-Naggar M.E. (2014). Antimicrobial Wound Dressing and Anti-Inflammatory Efficacy of Silver Nanoparticles. Int. J. Biol. Macromol..

[61] Ferdous Z., Nemmar A. (2020). Health Impact of Silver Nanoparticles: A Review of the Biodistribution and Toxicity Following Various Routes of Exposure. Int. J. Mol. Sci..

